# An All-In-One Multifunctional Touch Sensor with Carbon-Based Gradient Resistance Elements

**DOI:** 10.1007/s40820-022-00875-9

**Published:** 2022-06-14

**Authors:** Chao Wei, Wansheng Lin, Shaofeng Liang, Mengjiao Chen, Yuanjin Zheng, Xinqin Liao, Zhong Chen

**Affiliations:** 1 Department of Electronic Science, Xiamen University, Xiamen, 361005 People’s Republic of China; 2 Innovation Laboratory for Sciences and Technologies of Energy Materials of Fujian Province, Xiamen, 361005 People’s Republic of China; 3 School of Electrical and Electronic Engineering, Nanyang Technological University, Singapore, 639798 Singapore

**Keywords:** Multifunctional touch sensor, Carbon functional material, Paper-based device, Gradient resistance element, Human–machine interaction

## Abstract

**Highlights:**

- Carbon-based gradient resistance element structure is proposed for the construction of multifunctional touch sensor, which will promote wide detection and recognition range of multiple mechanical stimulations.
- Multifunctional touch sensor with gradient resistance element and two electrodes is demonstrated to eliminate signals crosstalk and prevent interference during position sensing for human–machine interactions.
- Biological sensing interface based on a deep-learning-assisted all-in-one multipoint touch sensor enables users to efficiently interact with virtual world.

**Abstract:**

Human–machine interactions using deep-learning methods are important in the research of virtual reality, augmented reality, and metaverse. Such research remains challenging as current interactive sensing interfaces for single-point or multipoint touch input are trapped by massive crossover electrodes, signal crosstalk, propagation delay, and demanding configuration requirements. Here, an all-in-one multipoint touch sensor (AIOM touch sensor) with only two electrodes is reported. The AIOM touch sensor is efficiently constructed by gradient resistance elements, which can highly adapt to diverse application-dependent configurations. Combined with deep learning method, the AIOM touch sensor can be utilized to recognize, learn, and memorize human–machine interactions. A biometric verification system is built based on the AIOM touch sensor, which achieves a high identification accuracy of over 98% and offers a promising hybrid cyber security against password leaking. Diversiform human–machine interactions, including freely playing piano music and programmatically controlling a drone, demonstrate the high stability, rapid response time, and excellent spatiotemporally dynamic resolution of the AIOM touch sensor, which will promote significant development of interactive sensing interfaces between fingertips and virtual objects.
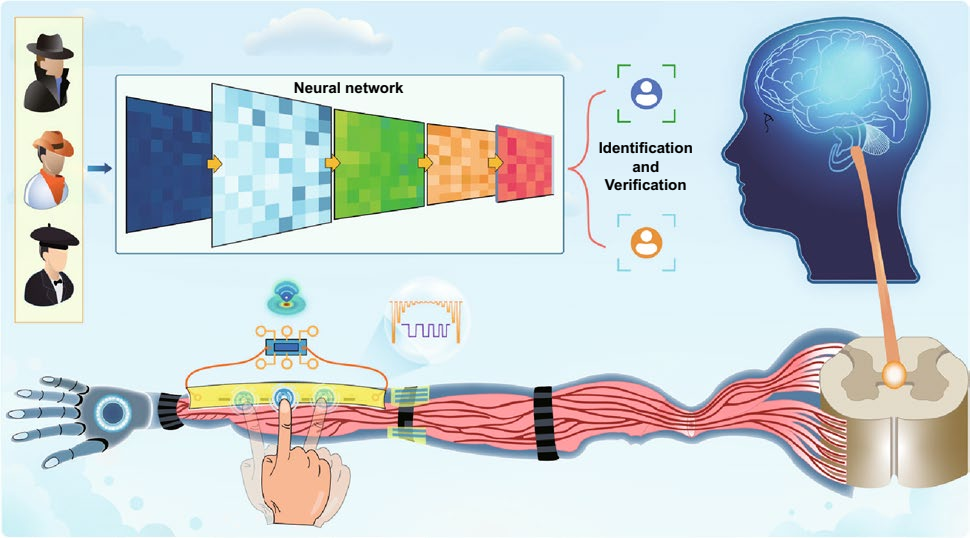

**Supplementary Information:**

The online version contains supplementary material available at 10.1007/s40820-022-00875-9.

## Introduction

A growing trend for human–machine interfaces (HMIs) hinges on the progress of a full-body immersive experience [1–3]. HMIs are medium to transmit and exchange information between human and machines for the accomplishment of a defined task [4–6]. Touch sensors are one of the important HMIs that can be created for diverse interactions between our body and virtual or real world. Owing to the advancement of artificial intelligence [7, 8], intelligent cyber security design with HMIs has become a remarkable application in financial and computing industry, which requires touch sensors to function in conjunction with deep learning. For a touchpad, single-point touch input enables simple control, such as tap, swipe, and drag, and multipoint touch input enables skillful operation identification for image scaling and object control. Touch sensors evolve from a single function with simple structure to multiple functions with high-resolution array structure, and their current trend is to achieve multiple functions based on ingenious design. Nowadays, electronic programmable touch sensors as interactive platforms for virtual reality (VR) [9, 10], augmented reality (AR) [11, 12], and metaverse are restricted to the limited function with instability and signal interference and the intricate structure with large number of electrodes.

To simultaneously monitor and recognize multipoint mechanical stimuli, researchers have explored various touch sensors. These electromechanical touch sensors for human–machine interactive systems lean upon different transduction mechanisms, including resistance [13–15], capacitance [16–18], triboelectricity [19, 20], piezoelectricity [21, 22], etc. These representative sensing mechanisms based on various materials and design configurations improve the performance of touch sensors. System-level integration with touch sensors for human–machine interactive applications, such as wearable electronic devices for object control [23], robotics [24], prosthetics [25], and VR/AR learning environment design [26], is explored while the progress is limited. Note that touch sensors generally consist of a sensing elements array with *M* × *N* × 2 electrodes array, where *M* and *N* represent the longitude and latitude lines, to accurately recognize the position of mechanical stimuli [27]. Through the system structure design, a touch sensor can significantly reduce the number of electrodes to *M* × *N* + 1 electrodes array or *M* + *N* electrodes array by performing a cross scanning addressing mode [28–30], which greatly shortens the measurement period, simplifies the process of signal processing and analysis, and reduces system configuration requirements. Minimalistic touch sensor design with a smaller number of electrodes will be benefit with lower signal crosstalk and faster signal detection, higher stability and lower system configuration requirements for human–machine interactive systems. Carbon-based materials have a wide range of application prospects as advanced materials [31–33], which can be served as the functional sensitive materials of touch sensors.

In this work, we propose an all-in-one multifunctional touch sensor (AIOM touch sensor), which is inspired by the biological perception, fusion, transmission, and recognition of mechanical stimulations [34], for human–machine interactive systems. Our AIOM touch sensor is adopted a novel structure based on carbon material, which is called as carbon-based gradient resistance elements that enable multipoint touch control with only two electrodes. In this way, signals crosstalk is eliminated and system configuration requirements with multitudinous electrodes are dramatically reduced. As a proof-of-concept, we explore the gradient resistance elements structure of AIOM touch sensor based on pencil-drawn graphite on paper, which is an efficient, convenient, and facile way and will be extended by printing methods of other functional composite materials. The functional graphite patterns can be turned into user-defined intriguing and vivid multifunctional touch sensor, which offers high stability, fast response time and excellent spatiotemporally dynamic resolution. To increase more functions, the AIOM touch sensor combined with machine learning offers the opportunities for performing intricate assignment. An artificial intelligence-assisted biometric verification system based on the AIOM touch sensor and a deep-learning network algorithm is constructed to classify and recognize inherent users’ behavior of human–machine interactions. It provides a promising cyber security layer against password leaking and network attacks for the protection of personal information. In addition, the AIOM touch sensor can be functionalized as a linear interactive control interface or a circular touch panel for VR/AR interactions, such as freely playing piano music and programmatically controlling a drone. This work not only demonstrates a new strategy to drive breakthrough developments in touch sensors, but also provides a scientifically and technically feasible way of thinking to build efficient and multifunctional touch sensors, which will benefit information communication, industrial productivity, clinical medicine monitoring, and metaverse era.

## Experimental

### Fabrication of the AIOM Touch Sensor

First, four pieces of adhesive tape (Scotch MagicTM Tape 810#, 3 M Inc.) were pasted on graph paper (No.704, QIANCAILE) to form a specified closed hollow pattern. Among them, two pieces of adhesive tape were parallel to the upper and lower on the graph paper, which were pasted with an interval of 5 mm. Other two pieces of adhesive tape were parallel to the left and right on the graph paper, which were pasted with an interval of 10 mm. Generally, the hollow area was 5 × 10 mm^2^. Subsequently, an 8B pencil (No. 6841, Deli Company) was then adopted to draw on the hollow area of the graph paper. Several times of drawing was performed to make the hollow area fully filled with graphite. After peeling off a layer of the tape-based pattern, the smooth and even graphite film would be formed as a predominant conductive path. The resistance value of graphite film with a fixed area could be changed as needed by repeated pencil drawing or scraping. During preparation, real-time resistance testing was performed through a multimeter until achieving the resistance value we need. Subsequently, the gradient resistive elements on the bottom layer were designed by drawing different graphite content on the corresponding hollow area. Furthermore, each gradient resistance element was spaced 10 mm apart. After that, to form the seamless connection between the gradient resistance elements, a sliver circuit scribe pen (Ink resistance of 1 Ω cm^−1^, Bare Conductive Inc.) was drawn on the spacing of gradient resistance elements, which formed the sliver conductive film. The sliver conductive film area was 2 × 10 mm^2^. The sliver conductive film was drawn multiple times by using the sliver circuit scribe pen, which controlled the resistance of the sliver conductive film close to 1 Ω cm^−1^. Then, the sliver circuit scribe pen was drawn inwards, along both ends of the gradient resistance elements. These two silver conductive films paralleled to the gradient resistance elements were 2 × 4.5 mm^2^, which ensured the 1 mm interval to hold disconnected. Afterward, the active touch buttons were designed to short-circuit the gradient resistance elements. The active touch buttons were mainly composed of silver conductive films separated from the upper and bottom layers, which were placed above the extension cord of the bottom silver conductive film. The active touch buttons were 10 × 10 mm^2^, and the sliver conductive films on the active touch button were 2 × 2 mm^2^. The size of the silver conductive film on the upper active touch buttons was larger than the interval on the bottom layer, which ensured that the gradient resistance elements on the bottom layer were short-circuited. Subsequently, the double-sided tape (No. 5000NSWH, NITTO Inc.) was pasted on both sides of the interval to fix the active touch button, which served as the spacer. The typical thickness of the spacer was 0.16 mm. The left and right ends of the graphite film were the positions of two electrodes of the AIOM touch sensor. Two conductive lines were respectively fixed at the positions of two electrodes by silver paint (SPI-PAINT, Structure Probe, Inc.). The above step fabricated a basic structural section of the AIOM touch sensor. The images of the typical AIOM touch sensor were shown in Fig. S6. Finally, the AIOM touch sensors were assembled by taping identical sized graph paper face to face, using the adhesive tape, to prevent the graphite from changing due to unintentional touching.

### Signal Process and Analysis

The microcontroller unit (MCU) used in this design was Arduino Leonardo, which was a microcontroller board based on ATmega32u4. It contained 20 digital input/output pins (7 for pulse width modulation (PWM) output, 12 for analog input), a 16 MHz crystal oscillator, a micro universal serial bus (USB) interface, a direct current (DC) interface, an in circuit serial programmable (ICSP) interface, and a reset button. Unlike all previous Arduino controllers, Arduino Leonardo used the USB communication function of the ATmega32u4 directly, eliminating the process of USB to universal asynchronous receiver transmitter (UART) chip, which allowed Leonardo to connect to the computer not only as a virtual cluster communication (COM) port, but also as a mouse or keyboard. An auxiliary resistor in series with the AIOM touch sensor was added to the circuit for voltage distribution, which was supplied by MCU with 5 V. The *A*_0_ pin for the MCU analog input port connected to the node between the AIOM touch sensor and the auxiliary resistor. When the external mechanical stimulation was applied to one active touch button or multiple active touch buttons of the AIOM touch sensor, the response resistance of the AIOM touch sensor was generated. Then, the sampled voltage ranging from 0 to 5 V, appearing at the analog input pin *A*_0_, was digitized into the range of 0–1023 by the ADC. The MCU generated different analyses and judgments according to the change of back-end action by logic operation. After the normalization value and threshold comparison, each action instruction corresponded to each set of digital signals in human–computer interaction applications. The command reached the communication network between the microcontroller and the target object by USB to manipulate objects with the pre-defined instructions in the application.

### Characterization and Measurement

For a clear observation, field emission scanning electron microscope (FESEM, SUPRA55 SAPPHIRE) and energy dispersive spectrometer (EDS, X-MAX80 Oxford) were utilized to observe the surface micromorphology and analyze the elemental composition of the graphite film with different carbon content, the silver conductive ink film, and graph paper. The digital optical microscope (ZWSP-4KCH, Medium Micro Innovation Technology Co., Ltd.) was employed to observe the coating uniformity of rich graphite, medium graphite, rare graphite and silver conductive ink. The Raman spectra of the samples were obtained by Raman Spectroscopy instrument (lDSPeC ARCTlC). The output voltages were measured with an oscilloscope (Gwinstek MFG-2220HM). The response resistance of the AIOM touch sensor was detected and recorded by digital multimeters (Proskit MT-1236 and Keysight 34465A). The current–voltage curves were measured by a semiconductor characterization system (Keithley 2400). Furthermore, the dynamic pressure was generated by the customized actuator (Beijing Times Brilliant Electric Technology Co., Ltd.) applied on the AIOM touch sensor for the tests. The thickness of the spacer was measured by the vernier caliper (K20K271592, GuangLu Digital measurement and control Technology Co., Ltd.). The standard force sensor (Bengbu Sensors System Engineering Co., Ltd., JHBS-1000G) was used to measure the corresponding finger press applied force for quantified measurement.

## Results and Discussion

### Operation Principle and Design of AIOM Touch Sensor

**Fig. 1:**
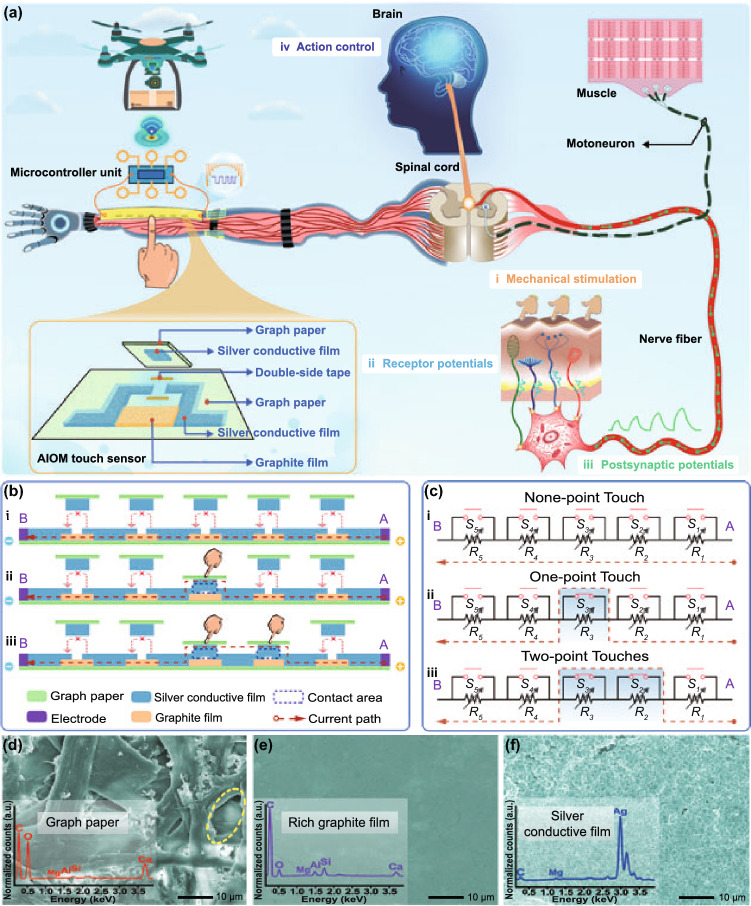
Architecture of the AIOM touch sensor for multipoint touch interactions. **a** Illustration of the AIOM touch sensor and its conceptual human–machine interactive application (Left), comparing with human sensory system (Right). **b**–**c** Working mechanism and corresponding equivalent circuits of the AIOM touch sensor for none, one, two mechanical stimulations, which were presented in the front view. **d–f** FESEM images and EDS spectrums of the graph paper, rich graphite film, and sliver conductive film

The detailed internal structure of the AIOM touch sensor, of which the working mechanism was inspired by the functions of the biological sensory neural system, was presented in Fig. 1a. Biologically, multiple fingertips touches, which are equal to multiple external mechanical stimulations, on the skin layer will be converted into transient receptor potentials by multiple mechanoreceptors, such as Meissner corpuscle (MC), Ruffini corpuscle (RC), Merkel disk (MD), and Pacinian corpuscle (PC) (the bottom right corner in Fig. 1a) [35]. The receptor potentials will make biological synapses to release neurotransmitters, which induce neuronal effector target cells to generate postsynaptic potentials. Subsequently, the postsynaptic potentials are transmitted along nerve fibers and the spinal cord to the brain. Finally, the brain will decode the received signals for analysis and judgment, and make a feedback action on these signals, such as promoting the motoneuron to innervate the skeletal muscles for a movement (the top right corner in Fig. 1a). In this biological sensory neural system, there are several core components, including mechanoreceptors to convert external mechanical stimulation into receptor potentials, biological synapses to induce postsynaptic potentials, nerve fibers to fuse and transmit postsynaptic potentials to the central nervous system, and the brain to analysis, judgment, and make a decision. Similarly, common touch sensing systems are also composed of many components, including many sensitive units, a corresponding large number of electrodes, signal conversion units array, etc., to achieve the functions of multipoint touch perception, conversion, transmission, and recognition. Obviously, the integration of this approach will cause a series of adverse consequences, such as complex and cumbersome system construction process, slow signal detection and recognition capabilities, severe signal interference, and high system configuration requirements.

In our design, the AIOM touch sensor based on two electrodes and gradient resistance elements could efficiently realize the functions of multipoint touch control, and a microcontroller unit was connected to the AIOM touch sensor to make corresponding actions for user-defined human–machine interactive applications (the top left corner in Fig. 1a). Note that the functions of the AIOM touch sensor are the same as the common touch sensors constructed with a complex structure, while the AIOM touch sensor technically avoids the unfavorable factors of common touch sensors. As a proof-of-concept, graphite, silver conductive film, and paper were the main raw materials used to prepare the gradient resistive elements and active touch buttons since they were easily prepared into diversified shapes and structures [36–38]. A representative structure of the AIOM touch sensor with one gradient resistance element was presented at the bottom left corner in Figs. 1a and S1. In the preparation of the materials, the resistance range of graphite film was in the kilo-ohm level, while the resistance of silver conductive film was relatively small in the range of several ohms (Fig. S2). The active touch buttons were mainly composed of silver conductive films and paper separated from the upper and bottom layers. The bottom layer of silver conductive film was mainly connected to the graphite film as a signal transmission channel. The silver conductive film on the upper layer was mainly used as a touch switch function to determine whether a graphite film was short-circuited. To make the upper and lower layers of the active touch button contact close, the touch pressure should be larger than about 5 kPa. In daily life, the pressure value was relatively small. A gentle finger press could exceed this value. Therefore, the total resistance (*R*_total_) of the AIOM touch sensor was mainly affected by the gradient resistance elements, which were graphite films. When no external mechanical stimulation was applied on the AIOM touch sensor, the current passed directly through the graphite film and the silver conductive film on the bottom layer. When touching the active touch button that brought the upper and bottom silver conductive film into close contact, the graphite film would be short-circuited as the resistance of the silver conductive films was much smaller than that of the graphite film. Therefore, the total resistance of the AIOM touch sensor would be changed so that the touch actions could be recognized according to the change in resistance.

The simplified front section view structure of the whole AIOM touch sensor with five gradient resistance elements was shown in Fig. 1b, where the current path was displayed from the positive electrode through the graphite films and the silver conductive films to the negative electrode. The corresponding equivalent circuits of the working mechanism of the AIOM touch sensor were illustrated in Fig. 1c, where the switches and the resistance units corresponded to the active touch buttons and graphite film, respectively. When no finger touching on the AIOM touch sensor that all the upper and bottom silver conductive films were separated, the current passed directly through all the graphite films and the bottom sliver conductive films (Fig. 1bi).

In the situation, all the switches were in an open state (Fig. 1ci). The total resistance of the AIOM touch sensor was almost equative to the sum of the resistance of all the graphite films, which was expressed as *R*_total_ = *R*_1_ + *R*_2_ + *R*_3_ + *R*_4_ + *R*_5_, where the *R*_1_, *R*_2_, *R*_3_, *R*_4_, and *R*_5_ were the resistance of different graphite films. After one finger touching on an active touch button of the AIOM touch sensor, such as the third one, the corresponding upper and bottom silver conductive films of the active touch button would be contacted, generating a mechanosensitive signal from the AIOM touch sensor (Fig. 1bii). Since the resistance of the silver conductive film was much smaller than that of the graphite film, the active touch button composed of the upper and bottom silver conductive films was equivalent to the switch *S*_3_ that could short the corresponding graphite film (*R*_3_) (Fig. 1cii). In this case, the current path would be changed in response, which passed through the resistance units *R*_1_ and *R*_2_, the switch *S*_3_, and the resistance units *R*_4_ and *R*_5_. Eventually, the resistance of the AIOM touch sensor turned as *R*_total_ = *R*_1_ + *R*_2_ + *R*_4_ + *R*_5_, where the third graphite film was shorted. In other situation, when two finger touches made two active touch buttons (the second and third buttons) of the AIOM touch sensor contact, a different mechanosensitive signal would be generated from the AIOM touch sensor as the current passed through these two active touch buttons instead of the corresponding graphite films (Fig. 1biii). Correspondingly, the other switches remained open and the switches *S*_2_ and *S*_3_ closed to short the resistance units *R*_2_ and *R*_3_ (Fig. 1ciii). Therefore, the resistance of the AIOM touch sensor was expressed as *R*_total_ = *R*_1_ + *R*_4_ + *R*_5_. The sensing mechanism of other multipoint touches, such as three-point touches, four-point touches, and five-point touches, was similar to that of two-point touches. When each additional touch point increased, the corresponding active touch button was pressed. In general, when multiple active touch buttons were pressed, the corresponding response resistance of the AIOM touch sensor was expressed as:

1 $$ R_{\text{total}} = R_{\text{all}} - \sum R_{i} $$

where *R*_all_ was the initial resistance of the AIOM touch sensor and the resistance (*R*_*i*_) of each gradient resistance elements were shorted by the corresponding switch *S*_*i*_. Therefore, external mechanical stimulations no matter one-point touch or multipoint touches could be efficiently converted into the electrical signals and the total resistance of the AIOM touch sensor varied with the number and position of external mechanical stimulations. Depending on the change in the response mechanosensitive signals, the AIOM touch sensor could recognize the different positions of external mechanical stimulations and their number. Overall, the AIOM touch sensor endowed the integrated functions, including conversion and recognition of multiple mechanical stimulations and transmission of mechanosensitive signals, for multipoint touch operation. As the resistive principle was relatively simple read-out and the corresponding devices was easy to be constructed. Therefore, the AIOM touch sensor was based on the resistive principle in this work. Regarding other principles, we think of other principles as well as the possibility of achieving multi-touches recognition with only two electrodes based on similar gradient elements. The relevant work based on other principles needs to be further designed and validated in the future.

Carbon pencil was an artificial nanocomposite containing conductive graphite and clay [14, 39]. Pencil-carbon material was easy to use and low-cost. The graphite film could be easily deposited on a graph paper substrate by a carbon pencil during writing or drawing process. Writing and drawing by pencil, several electronic devices, including strain sensors [39] and pressure sensors [14], have been fabricated to be freely designed as needed. For the rapid preparation of the prototype AIOM touch sensor, the method of pencil drawing was employed. In this work, the method of pencil drawing, owning a fast and efficient advantage compared with photolithography, molecular beam epitaxy, and chemical vapor deposition, was chosen to prepare the gradient resistance elements. Field emission scanning electron microscopy (FESEM) images of the adopted graph paper, the graphite film with rich carbon content, and sliver conductive film were obtained to characterize the structural features of the materials (Fig. 1d–f). It could be observed that the morphology of the graph paper was porous, which was composed of cellulose fibers by cross stacking, as shown in the yellow dotted ellipse in Fig. 1d. The porous structure of the graph paper facilitated the deposition of graphite film by pencil drawing. The energy dispersive spectroscopy (EDS) image of the graph paper (inset spectrum in Fig. 1d) indicated that the main elements of the graph paper were carbon and oxygen, which were the main elements of the cellulose fibers. The flat graphite films with different resistances, as the gradient resistance elements, were obtained easily by pencil repeatedly drawing on the graph paper. Three typical FESEM images of the graphite film showed the rare graphite film (Fig. S3a), medium graphite film (Fig. S3b), and rich graphite film (Fig. 1e), which represented graphite films with different carbon content from low to high. Their EDS images (inset spectra in Fig. S3a−b and inset spectra in Fig. 1e) demonstrated that the higher carbon content of graphite film, the higher its carbon element and the lower its oxygen element. For connecting the graphite films, a sliver circuit scribe pen drew several times on the graph paper to obtain the silver conductive film. The inset EDS spectrum in Fig. 1f indicated that the silver conductive film contained a large amount of silver elements. A strong connection between the graphite film and the silver conductive film could provide basic support for the stable operation and effect realization of the gradient resistance elements with graphite films (Fig. S3c–d). The microscopic morphology characterizations (Fig. S3e–f) were also carried out to show the clear boundary between the silver film and the graph paper, which ensured that the conductive path could be designed efficiently according to the needs of the device’s structure and function. In addition, the Raman spectra of rare graphite film, medium graphite film, and rich graphite film indicated the characteristic peaks of different graphite content at the same location and three prominent peaks at 1350, 1579, and 2700 cm^−1^ corresponding to the D, G, and 2D bands of carbon, respectively (Fig. S4), which were also the characteristic peaks of graphene. The graphite films were resistive-type elements and the resistance of the graphite films with the different carbon content was different (Fig. S5). It should be noted that the resistance of the graphite film could be effectively modulated by pencil repeatedly drawing on the graph paper and eraser removing some of the graphite, which provided the basis for preparing on-demand gradient resistance elements.

### Performance and Characteristic of the AIOM Touch Sensor

**Fig. 2:**
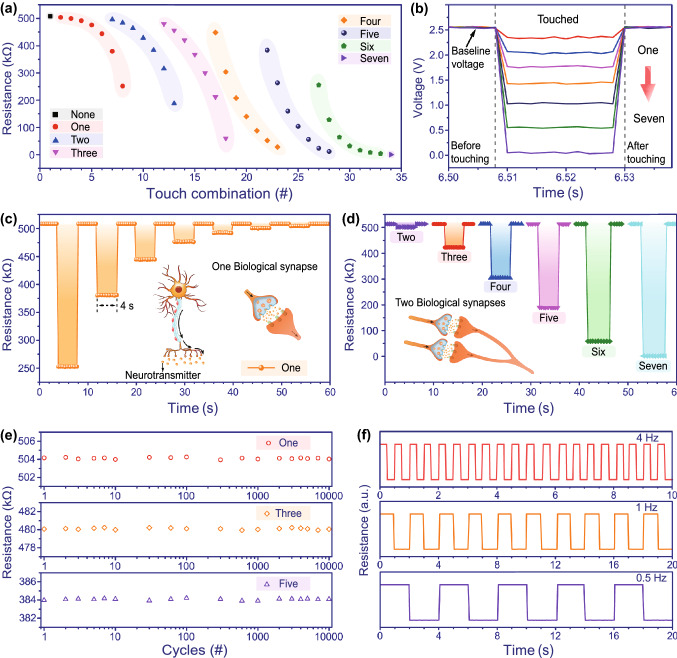
Performance and characteristic of the AIOM touch sensor. **a** Diagram of the typical trend of the response resistance for touching different active touch position with different touch points. **b** Response time test of the AIOM touch sensor. External mechanical stimulations were applied to the AIOM touch sensor from first to seventh active touch buttons. **c** Spatiotemporally dynamic response of the AIOM touch sensor based on one-point touch. Inset image: Schematic of neuron with neurotransmitter and a biological synapse. **d** Spatiotemporally dynamic response of the AIOM touch sensor based on different combination of multiple touches ranging from two-point touches to seven-point touches. Inset image: Schematic of spatiotemporally dynamical mode and information fusion of two biological synapses. **e** Stability of the AIOM touch sensor for more than 10,000 times of cyclical touching-removing tests applied by different touch combinations. **f** Response of the AIOM touch sensor under cyclically touching with frequencies of 0.5, 1, and 4 Hz for two-point touches

To characterize and analyze the mechanosensitive performance of the AIOM touch sensor, we constructed the AIOM touch sensor with seven gradient resistance elements, where the resistance of the graphite films was 4, 8, 16, 32, 64, 128, and 256 kΩ, respectively (Fig. S6). The resistance (*R*_*i*_) of each gradient resistance element was designed using this formula: *R*_*i*_ = 2^*i*−1^ × *R*_*1*_, where the *R*_*1*_ and *i* were the resistance of the first gradient resistance element and the serial number of the gradient resistance element, respectively. Based on experimental experience, the resistance of the first gradient resistance element was selected to be 4 kΩ for the proof of concept. By touching the active touch buttons, the AIOM touch sensor would produce the corresponding response resistance (Fig. 2a). The response resistance corresponded to the touch positions and the number of the touch points (the active touch buttons). Due to the structural design based on the gradient resistance elements, the resistance of the resistance elements was different from each other. In Fig. 2a, when the numbers of touch points were zero and seven, the response resistances of the AIOM touch sensor were around 508 kΩ and 30 Ω, respectively. When one active touch button was pressed, the response resistance of AIOM touch sensor was about 504, 500, 492, 476, 444, 380, or 252 kΩ, respectively. As a typical example of the multipoint recognition, when three active touch buttons were pressed, the response resistance of the AIOM touch sensor was about 480, 456, 420, 368, 300, 212, or 60 kΩ, respectively. The number of the response resistance of the AIOM touch sensor with seven gradient resistance elements could be up to 128 (Fig. S7), and there was a minimum interval resistance of 4 kΩ between the different response resistance of the AIOM touch sensor. When the numbers of touch points were zero and seven, the response resistances were the highest and lowest value, respectively. When the number of touch points was one, two, three, four, five, or six, the response resistance varied as the touch combinations. In the test, the touch points started with the gradient resistance elements of small resistance value. For example, when the touch point was only one point, the gradient resistance elements with a resistance value of 4, 8, 16, 32, 64, 128, or 256 kΩ were successively shorted by touch. Therefore, the resistance value of the AIOM touch sensor decreased according to the corresponding resistance value. When the touch combination was based on two points, the test was also conducted according to shorting the gradient resistance elements from a small resistance value to the gradient resistance elements with a large resistance value. Therefore, corresponding to different touch combinations, the resistance value of the AIOM touch sensor would decrease gradually. With the increasing number of touch points, the response resistances were in an overall decreasing trend. Since each touch case (one-point touch or multipoint touches with different touch positions) would correspond to a different response resistance, the number of the response resistance (*N*_r_) of the AIOM touch sensor responding to all touch cases could be expressed as the following equation:

2 $$ N_{{\text{r}}} = \mathop \sum \limits_{i = 1}^{n} C_{n}^{m} = \mathop \sum \limits_{i = 1}^{n} \frac{n!}{{m!\left( {n - m} \right)!}} = \mathop \sum \limits_{i = 1}^{n} \frac{{n\left( {n - 1} \right) \cdots \left( {n - m + 1} \right)}}{{m\left( {m - 1} \right) \cdots 1}} $$

where *C*_*n*_^*m*^, *n*, and *m* were the combination of touches, the number of the gradient resistance elements, and the number of the touch points, respectively. Thus, the number of the response resistance of the AIOM touch sensor with seven gradient resistance elements could be up to 128 (Fig. S7), which reflected the functional versatility of the design scheme. The result indicated that the response resistance was highly regional differentiated to all touch cases. Therefore, the AIOM touch sensor based on the structure of two electrodes and the gradient resistance elements could fully recognize different touch positions and different number of touch points. Furthermore, the structure of the AIOM touch sensor was extensible. Depending on the needs of interactive applications, the active touch buttons and the gradient resistance elements could be adjusted appropriately, which could be increased or decreased. It would promote wider detection and recognition range of mechanical stimulations and provide richer possibilities and practicability for artificial intelligence-assisted human–machine interactive applications.

Since there was a response time for the AIOM touch sensor to change state before and after touching, the response time was evaluated (Fig. 2b). There was a baseline voltage that represented the standby status of AIOM touch sensor before touching. We conducted pre-stimulus and post-stimulus response tests for all the one-point touch and multipoint touches to demonstrate the feasibility of the AIOM touch sensor. The response time for the transition state before and after touching was < 3 ms, which far surpassed the average person's reaction time (~ 200 ms) and was excellent to meet rapid detection requirements.

In biological sensory system, spatiotemporally dynamic stimulations converted by different mechanoreceptors could trigger synapses to induce postsynaptic potentials and establish a dynamic logic in the neural network. Analogously, the comparison and analysis of mechanosensitive signals of the AIOM touch sensor corresponding to different numbers and positions of touches could evolve into a dynamic behavioral decision. When only one dynamic mechanical stimulation was applied to the AIOM touch sensor, the transmission of signals was analogous to the transmission of the sensory neural system. When the mechanical stimulation was applied to the AIOM touch sensor, the AIOM touch sensor would become excited, which was reflected by the mechanosensitive signal occurring. After removing the mechanical stimulation, the mechanosensitive signal of the AIOM touch sensor returned to the original state (Fig. 2c). No matter which one active touch button was touched with the lasting time of 4 s in a dynamic test, the response of the AIOM touch sensor was in a regular fluctuation, which was similar to the excitation and inhibition of a biological synapse. In addition, it could be found that the mechanosensitive signals of the AIOM touch sensor were stable to respond to the mechanical stimulation. To represent the round-trip identification of the AIOM touch sensor in the process of mechanosensitive signal transmission, we designed the process of touching the active touch buttons forth and back from first to seventh active touch buttons with the lasting time of 4 and 1 s (Fig. S8a-b). The results demonstrated that the AIOM touch sensor had high robustness and stability in operation. The energy consumption (*W*) of the AIOM touch sensor could be calculated using this formula: *W* = *U*^2^/*R*, where the *U* and *R* were the applied voltage and the resistance of the AIOM touch sensor, respectively. Analogous to the transmission of neurotransmitters through a biological synapse (inset image in Fig. 2c), when the voltage kept constant, the energy consumption would be very low if the resistance was in a high level. The AIOM touch sensor featured relatively low energy consumption in the standby state of no mechanical stimulation, which was similar to the ineffective in excitation during neurotransmission with few neurotransmitters. Subsequently, when the mechanosensitive signals of the AIOM touch sensor occurred, reflected in the rapid decrease of the resistance, the energy consumption increased.

Excepting the recognition of spatiotemporally dynamic mechanical stimulation based on different one-point touch position, the AIOM touch sensor could recognize the information fusion of spatiotemporally dynamic mechanical stimulations based on multipoint touch positions. Biologically, different multipoint mechanical stimulations were converted by multiple mechanoreceptors. The two biological synapses (inset image in Fig. 2d) showed that the signal transmission generated from two-point touches was similar to the function of two biological synapses. Similarly, signal transmission generated from more-point touches meant that more synapses were needed to achieve functionality. Through multiple biological synapses, the information of mechanical stimulations was collectively transmitted into the sensory neural system, making an information fusion and then producing a spatiotemporally dynamic logic. For the function demonstration, multiple types of mechanical stimulations were simultaneously applied to different positions of the AIOM touch sensor (Fig. 2d). When two mechanical stimulations were simultaneously applied to the AIOM touch sensor, the fusional mechanosensitive signal was generated, like transmitting signals into the sensory neural system by two biological synapses. The response of the AIOM touch sensor was stable, and would turn to its original state after removing the mechanical stimulation. To assess a higher-order response of the AIOM touch sensor, more spatiotemporally dynamic mechanical stimulations were performed for the test. The fusional mechanosensitive signals were well-regulated and distinguishable to correspond one-to-one to different combinations of touches. In Fig. 2d, the two-point touches were located at touch positions 1 and 2. The three-point touches were located at touch positions 2, 3, and 4. The four-point touches were located at positions 1, 2, 5, and 6. The five-point touches were located at positions 1, 2, 3, 4, and 7. The six-point touches were located at touch positions 1, 2, 3, 4, 6, and 7. The seven-point touches were located at all touch positions. We also conducted round-trip touch tests with each lasting time of 1 and 4 s to verify the robustness of the multipoint touch function of the AIOM touch sensor (Fig. S8c-d). It could be found that the fusional mechanosensitive signals corresponding to the same combination of touches were almost in the same, indicating that the AIOM touch sensor had good stability and high robustness to convert, fuse, transmit, and recognize multipoint touches. The results further indicated that the mechanosensitive signals were highly regional differentiated to all cases of dynamic mechanical stimulations, and the AIOM touch sensor well realized the establishment and judgment of spatiotemporally dynamic logic with multiple combinations of touches. It should be noted that the AIOM touch sensor was only based on two electrodes, gradient resistance elements, and corresponding active touch buttons, which effectively avoided intricate interconnections and complex structures, and eliminated unnecessary interference in signal transmission and recognition.

To further test the stability of the AIOM touch sensor, three-type combinations of mechanical stimulations were applied to one, three, and five active touch buttons, respectively (Fig. 2e). More than 10,000 times of cyclical touching-removing tests were conducted. It was confirmed that the response resistance was almost unaffected, indicating that the AIOM touch sensor had perfect stability, robustness, and repeatability. Subsequently, we tested the response of the AIOM touch sensor by mechanical stimulations with different loading frequencies of 0.5, 1, and 4 Hz for two-point touches (Fig. 2f). The results showed that the dynamic response was continuous and stable for the changes of dynamic mechanical stimulations, which was similar to the excitation and inhibition of the sensory nerve. It was concluded that the AIOM touch sensor had the ability to accurately and continuously respond to almost all mechanical stimulations in daily life.

### Multifunctional Interactive Applications of the AIOM Touch Sensor

**Fig. 3:**
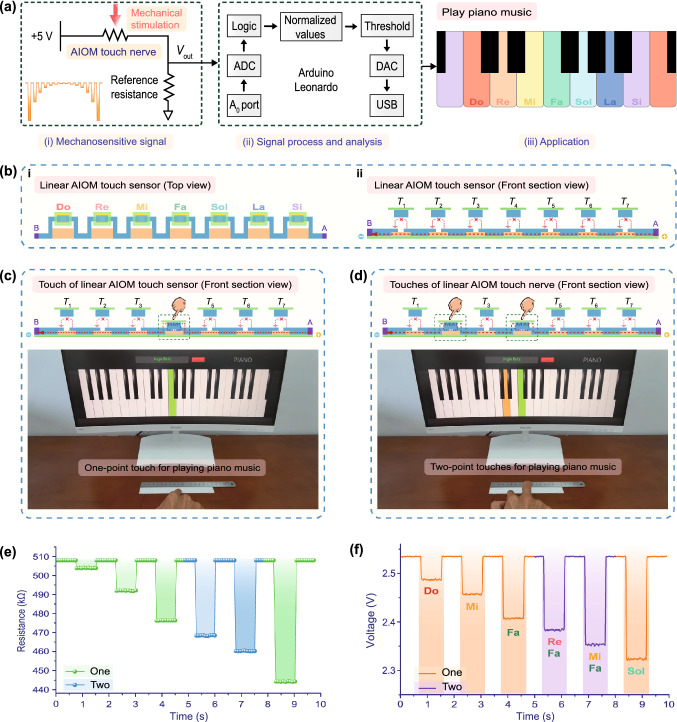
Linear AIOM touch sensor for freely playing piano music. **a** Circuit schematic to drive the linear AIOM touch sensor for conceptually playing piano music. **b** Schematic showing the linear AIOM touch sensor in (i) top view and (ii) front section view. **c** Schematic and photo of the linear AIOM touch sensor by one-point touch for playing piano music. **d** Schematic and photo of the linear AIOM touch sensor by two-point touches for playing piano music. **e** Response resistance of the linear AIOM touch sensor when touching one or two active touch buttons. **f** Variation in the voltage producing corresponding tone and harmony

As a conceptual application validation, a linear AIOM touch sensor was designed and constructed, which was served as a linear interactive control interface, for freely playing piano music (Fig. 3). When fingers touched on the active touch buttons of the linear AIOM touch sensor, mechanosensitive signals would be generated rapidly. Followingly, the signal process and analysis were performed by a microcontroller unit (MCU). Finally, the corresponding action commands would be transmitted to a computer for making a sound (Fig. 3a). The top view schematic of the linear AIOM touch sensor was shown to highlight the style that each active touch button was equivalent to a piano key (Fig. 3bi). The linear AIOM touch sensor with a working length of 120 mm contained a total of seven active touch buttons, from left to right, namely *T*_1_, *T*_2_, *T*_3_, *T*_4_, *T*_5_, *T*_6_, and *T*_7_, corresponding to the tone of Do, Re, Mi, Fa, Sol, La, or Si. When there was no touch, all the active touch buttons were in the open state and the linear AIOM touch sensor was in the standby mode (Fig. 3bii). When a finger touched one active touch button of the linear AIOM touch sensor, the corresponding mechanosensitive signal would be generated. Then, the virtual piano was activated to produce the corresponding tone (Fig. 3c). In addition, piano performances often had multiple keys working together to form a harmony. The above had proved that the AIOM touch sensor could recognize different combinations of multiple touches. Therefore, the combination diversity of different touches would be achieved by the linear AIOM touch sensor to form various harmonies. As an example, when simultaneously touching the *T*_2_ and *T*_4_ buttons of the linear AIOM touch sensor, a fusional mechanosensitive signal would be generated to respond to the action. The response resistance of the linear AIOM touch sensor was in the range from 467 to 469 kΩ. In the program, a signal within a certain range was set to correspond to a certain instruction, such as the resistance range from 467 to 469 kΩ corresponding to emitting the harmony of Re and Fa. After identifying and comparing the fusional mechanosensitive signal, the corresponding instruction would be sent to the computer. As a result, the virtual piano emitted the harmony of Re and Fa (Fig. 3d). When fingers touched one or two active touch buttons, the response resistance of the linear AIOM touch sensor was rapidly generated and maintained for a period of time as needed (Fig. 3e), while the output voltage of the driver circuit would be changed accordingly (Fig. 3f). As the changes in the output voltage were different from each other, the different combinations of touches could be switched freely. In such a way, we could touch multiple buttons in different positions to play more types of music (Fig. S9). A vivid human–machine interactive application of playing a piece of piano music by the linear AIOM touch sensor was presented in Movie S1, where playing not only one tone but also beautiful songs with chords.

**Fig. 4:**
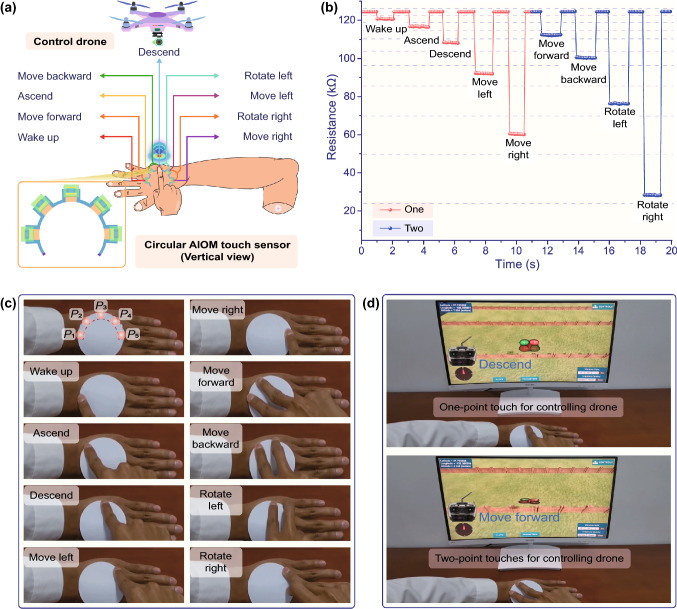
Circular AIOM touch sensor for programmatically controlling a drone. **a** A wearable control panel based on the circular AIOM touch sensor with five working points for controlling nine flight actions of a drone. Bottom left: Structure diagram of the circular AIOM touch sensor in top view. **b** Typical response resistances of the AIOM touch sensor corresponding to the control commands by one-point touch and two-point touches. **c** Photos of the specific combinations of the touches on the circular AIOM touch sensor related to the drone control commands. **d** Typical photos of the flight actions of a virtual drone by one-point touch and two-point touches on the circular AIOM touch sensor

Due to the scalability of the fabrication process and the versatility of the design principle, the AIOM touch sensor based on a circular structure could be designed and constructed as a circular touch panel for programmatically controlling a drone (Fig. 4a). The circular AIOM touch sensor was composited of two electrodes, five gradient graphite elements, and corresponding five active touch buttons with a disk with a diameter of 60 mm (the bottom left corner in Fig. 4a). As a proof-of-concept, the circular AIOM touch sensor could be attached to the back of the hand for controlling the drone. Each working point of the circular AIOM touch sensor was arranged in a ring from left to right and named as *P*_1_, *P*_2_, *P*_3_, *P*_4_, and *P*_5_. Since the circular AIOM touch sensor could identify both one-point touch and multipoint touches, it was possible to set up the combinations of one-point touch and multipoint touches for the programmatic interactive control of a drone. As a proof-of-concept, nine interactive commands were programmed based on the circular AIOM touch sensor for the flight actions of a drone, involving *P*_1_, *P*_2_, *P*_3_, *P*_4_, and *P*_5_ for “wake up”, “Ascend”, “Descend”, “Move left”, and “Move right”, respectively, and each combination of *P*_1_ and *P*_2_, *P*_2_ and *P*_3_, *P*_3_ and *P*_4_, *P*_4_ and *P*_5_ corresponding to “Move forward”, “Move backward”, “Rotate left”, “Rotate right”, respectively. When one-point touch or two-point touches were applied to the working points of the circular AIOM touch sensor, the response resistance would be generated rapidly (Fig. 4b). The response signal of the circular AIOM touch sensor could maintain stability for a period of time according to the need for flight actions. Once removing the fingertips touches from the active touch buttons, the response signal of the circular AIOM touch sensor rapidly returned to the original state. Since all the response resistances of the circular AIOM touch sensor differed from each other, the response resistance of the circular AIOM touch sensor was corresponding to the touch points in different positions and to multiple touch points in different positions at the same time. If the MCU received the resistance signals, the input information of touch point numbers/amounts could be reflected according to the response resistance of the circular AIOM touch sensor. Therefore, the MCU could easily identify them and made the virtual drone perform corresponding flight actions. Figure 4c showed the photos of the specific combinations of the touches related to the drone control commands. It could be found that the control of these interactive flight actions was relatively simple, clear, and facile. The typical virtual reality application of one-point touch and two-point touches for the flight actions of the drone was presented in Fig. 4d. A vivid control of a virtual drone by the circular AIOM touch sensor was displayed in Movie S2. In general, to control the nine flight actions of a drone, common devices requested to integrate nine distributed sensing elements with a mass of electrodes, which was a major concern for design space, manufacturing difficulty, and production period. The results demonstrated that the AIOM touch sensor containing only five active touch buttons could be designed to operate nine flight actions of the drone. In addition, more combinations of touches for programmable handling functions of the drone would be developed if needed (Fig. S10a-b). Moreover, the structural simplicity and functional versatility of the circular AIOM touch sensor was realized and effectively avoided the intricate interconnection and complex structures, as well as eliminated the interference in the signal transmission. In addition, as the back of the hand was not flat, the circular AIOM touch sensor would bend accordingly. The circular AIOM touch sensor with slight bending could still control a drone well. If using the same working principle and being replaced by other more flexible materials, the designed AIOM touch sensor may be better installed in any part of the body, and it needs to continue to research in the future.

### Deep Perceptual Learning Application of Biometrics Based on the AIOM Touch Sensor

**Fig. 5:**
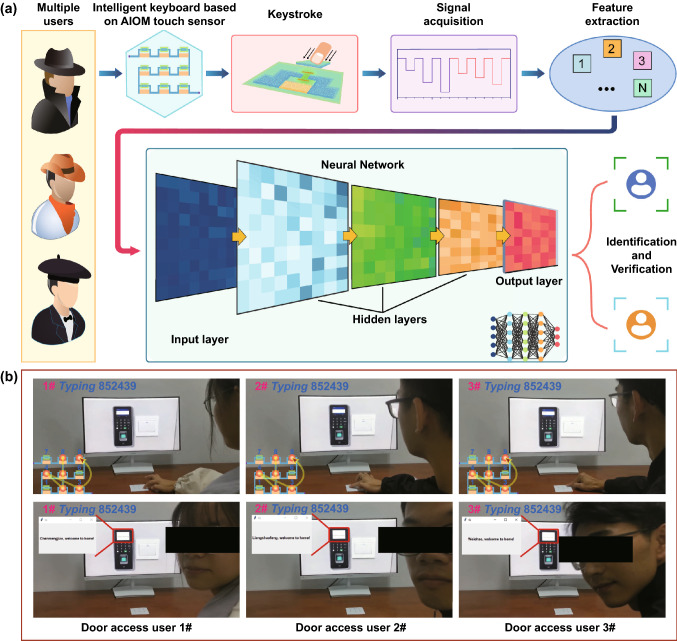
Augmented user verification system based on the AIOM touch sensor. **a** Schematic of an intelligent keyboard based on the S-typed AIOM touch sensor for user identification and verification. **b** Photos of three users typing the same password number of “852439” through the S-shaped AIOM touch sensor and each user being accurately identified by the ANN algorithm based on dynamic behavioral feature

The virtual reality application of biometrics for augmented user verification system is in increasing requirement of high cyber security and computer privacy security. Keystroke dynamics, which is one important sort of biometrics, captures user data with individual behavior primarily through different touch features, including touch positions, holding time, and intervals between touches. The S-shaped AIOM touch sensor with two electrodes, nine gradient graphite elements, and corresponding nine active touch buttons was designed and constructed as an intelligent keyboard of user identification and verification (Fig. 5a). Based on the S-shaped AIOM touch sensor, a keystroke dynamics of biometric approach assisted by artificial neural network (ANN) was proposed [40, 41]. ANN consisted of a neural network of interconnected processing neurons that mimicked the ability of the human brain to process complex linear and non-linear relationships using high-speed computing capabilities. First, the S-shaped AIOM touch sensor was mechanically stimulated by different users by touching, which allowed the continuous acquisition of users’ dynamic keystroke information in a non-invasive manner. Then, the raw mechanosensitive signals of the S-shaped AIOM touch sensor were acquired and converted into digital signals. Subsequently, the digital signals of the dynamic keystroke information were continuously processed through logical operations to extract the specific dynamic behavioral features. The extracted features were then transferred to the neural network for classification, which effectively identified the users’ dynamic behavior data and improved the accuracy of the keystroke behavioral recognition system. To build the training data set for the neural network model, we acquired 1800 times keystroke samples of three users and their dynamic behavioral features, mainly including three feature parameters of holding time, intervals, and signal magnitudes (reflecting touch positions). The signal processing flowchart of the ANN algorithm procedure for keystroke dynamics-based multiple users’ identification and verification based on the S-shaped AIOM touch sensor was shown (Fig. S11). This multi-classification ANN model, after being given a training set, extracted and classified the dynamic behavioral features of multiple touches, and then calculated the output of each network node in the neural network through the feed-forward back-propagation (BPP) algorithm for each training set. In practical application, the feed-forward BPP algorithm was mainly used in supervised learning technology of neural network due to its ability of general approximation and simple design [42]. The supervised learning provided the neural network with input data and output result data, and the weight was updated iteratively to reduce the difference between the actual output value and the expected output value. The feed-forward BPP algorithm consisted of the forward pass and the backward pass, both of which played a key role in accurate output results. In the forward pass, first, the input data was transmitted to the input layer in the network, then propagated along with the network through the weights and processing function of each neuron in the hidden layer. Subsequently, the output result was obtained in the output layer, and the error between the predicted value and the actual value was calculated to obtain the accuracy. In the backward pass, the error signals were sent from the output layer back to the input layer via the hidden layers, meanwhile, the weight and bias of each neuron were iteratively corrected to minimize the output error and achieve higher accuracy. Furthermore, the output of the final node of the neural network was compared with the desired output, and the errors were measured by a loss function of “Categorical Cross Entropy” [43]. The loss function, which assessed whether the algorithm procedure was satisfactory, would output a lower number during the data processing and indicate its accuracy. Since a prediction function was required in the deep learning application, the output layer of the algorithm procedure with the Softmax activation function was designed to calculate the weighted sum and bias in neural computing [44]. In the algorithm, the gradient descent optimization algorithm Adam (Adaptive moment estimation) with a first-order method was executed to achieve low time complexity [45]. The learning rate would become smaller as the parameter changed repeat, and the loss function was then minimized by adjusting the weights and biases. In the designed experiment of 600 keystroke samples per user, around 500 random samples were served as the training set through supervised learning, and the rest acted as the test set for user identification and verification.

As a proof-of-concept, a user access control system was built by the S-shaped AIOM touch sensor. Setting the 6-digits number sequence password number of “852439” as an example, a total of 17 features, involving 6 times of holding time, 5 intervals and 6 signal magnitudes, could be extracted. The main implementation method was that three users (Door access user 1#, Door access user 2#, Door access user 3#) typed the same password number of “852439”. The dynamic keystroke features of these typing actions were input to the ANN model to identify the user’s specific dynamic behavioral information and verified the user’s identity. Figure 5b described that three users typed the same password number of “852439” through the S-shaped AIOM touch sensor. In the lower left corner of the first row, the yellow dots on the keypad indicated the positions of touch. The order of the touch from 8, 5, 2, 4, 3 to 9 was indicated by a yellow arrow. It should be noted although three users typed the same password number, the keystroke dynamic information of each user was different because of different personal habits. To visually display mechanosensitive signals for the users’ dynamic behavioral features of multiple touches, each typing actions of three users were clearly recorded by the response resistance of the S-shaped AIOM touch sensor (Fig. S12). The result showed that the signal amplitude of each number is within a specific range, while the holding time and the intervals were totally different. According to the fact, each user could be accurately identified by the ANN algorithm. After that, the user access control system showed the corresponding information, e.g., “Chenmengjiao, welcome to home!”, “Liangshaofeng, welcome to home!”, or “Weichao, welcome to home!”. To better validate the robustness and accuracy of the ANN algorithm, the accuracy confusion matrix of the user access control system based on the S-shaped AIOM touch sensor with different user test datasets was shown in Fig. S13. Overall, the confusion matrices indicated that the ANN algorithm achieved an accuracy of over 98% for identification and verification. A dynamic process for testing the user access control system was vividly shown in Movie S3. By integrating artificial intelligence with the AIOM touch sensor, the user access control system could effectively learn, adapt, and identify information about the users’ keystroke behavior characteristics. The number of combinations of different touches was calculated according to the formula (2), so the password of the augmented user verification system could be set in a variety of ways according to the user's needs.

## Conclusions

We have reported the deep-learning-assisted AIOM touch sensor with only two electrodes, serving as a technologically feasible HMI for diversiform human–machine interactions between fingertips and virtual objects. Instead of M × N × 2 or M + N electrodes array, the minimalistic electrodes of the AIOM touch sensor prevented interference during sensing touch position and dramatically reduced the system configuration requirements for the conversion, fusion, transmission, and recognition of multipoint touches. The designed gradient resistance elements structure of the AIOM touch sensor would promote wide detection and recognition range of multiple mechanical stimulations and provide diverse possibilities and practicability for artificial intelligence-assisted human–machine interactions. The mechanosensitive signal of the AIOM touch sensor was highly regional differentiated to respond to different one-point touch position and multipoint touch positions of the spatiotemporally dynamic mechanical stimulations. The response time of the AIOM touch sensor for the transition state before and after touching was < 3 ms, which was much less than the average person's reaction time (~ 200 ms) and excellent to meet rapid detection requirements. The different one-point touch position and multipoint touch positions mechanical stimulations for single-trip and round-trip touch tests guaranteed the excellent robustness of the AIOM touch sensor. High response stability to three-type combinations of mechanical stimulations applied to multipoint active touch buttons against > 10,000 repetitive stimulations and the different touch frequencies was the other important characteristic of the AIOM touch sensor, which could make the AIOM touch sensor reliable without frequent signal corrections. In the light of its unique multipoint touch sensing structure and responding features, the AIOM touch sensor performed VR/AR applications, including freely playing piano and programmatically controlling the drone, which could effectively convert one or multiple dynamic mechanical stimulations into set commands. Based on the AIOM touch sensor with deep learning algorithm, the augmented user verification system could recognize, learn, and memorize different touch positions, holding time, and intervals of users’ keystroke behavior characteristics. Based on the identification and verification of users’ touch habits, the approach could achieve high accuracy of over 98%, demonstrating the promising cybersecurity layer against password vulnerability in the digital computing world. The AIOM touch sensor provided a progressive strategy in the process of multipoint touch excitation and inhibition of artificial tactile sensation computation, and would broadly provide an important tactile information confusion of sensing multiple mechanical stimulations for prosthetic design, health monitoring, robotics, and fantastic human–machine interaction application.

## Supplementary Information

Below is the link to the electronic supplementary material.

Supplementary file1 (AVI 2233 KB)

Supplementary file2 (AVI 8023 KB)

Supplementary file3 (AVI 12813 KB)

Supplementary file4 (PDF 1827 KB)
