# Supplementary material for: An All-In-One Multifunctional Touch Sensor with Carbon-Based Gradient Resistance Elements
Source: Nanomicro Lett. 2022 Jun 14;14:131. doi: 10.1007/s40820-022-00875-9 (PMC9198138; doi:10.1007/s40820-022-00875-9)
Supplement: Supplementary file 4 — Supplementary file4 (PDF 1827 KB) [file 40820_2022_875_MOESM4_ESM.pdf]

Supporting Information for

An All-In-One Multifunctional Touch Sensor with Carbon-Based Gradient Resistance Elements

Chao Wei[1], Wansheng Lin[1], Shaofeng Liang[1], Mengjiao Chen[1], Yuanjin Zheng[3], Xinqin Liao[1, 2, *], Zhong Chen[1, 2, *]

[1]Department of Electronic Science, Xiamen University, Xiamen 361005, P. R. China

[2]Innovation Laboratory for Sciences and Technologies of Energy Materials of Fujian Province, Xiamen 361005, P. R. China

[3]School of Electrical and Electronic Engineering, Nanyang Technological University, Singapore 639798, Singapore

*Corresponding authors. E-mail: liaoxinqin@xmu.edu.cn (Xinqin Liao), chenz@xmu.edu.cn (Zhong Chen)

Supplementary Figures

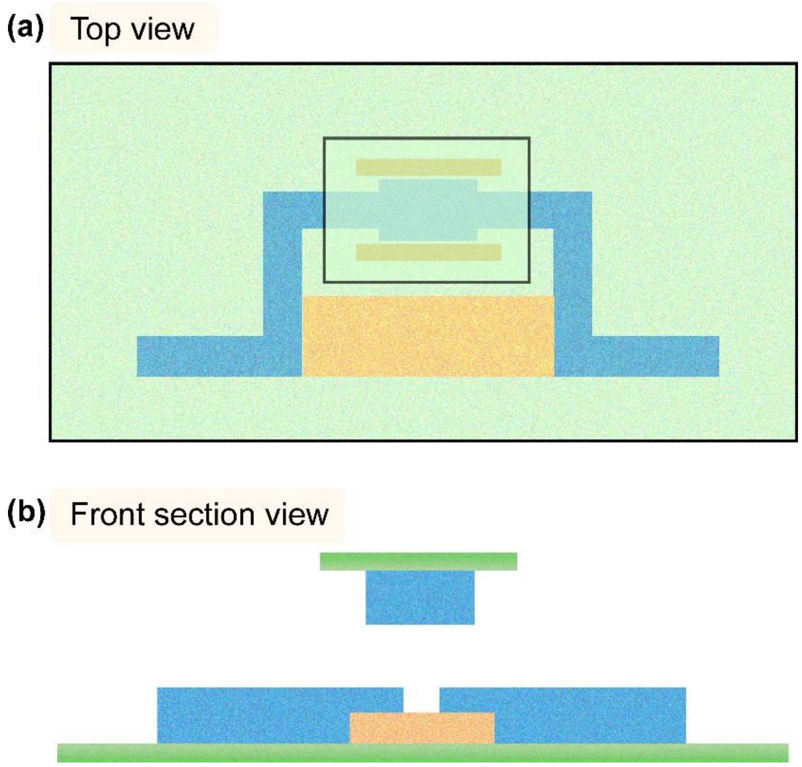

Fig. S1 Different azimuthal views of the AIOM touch sensor. **a** The structure of the AIOM touch sensor in top view. **b** The structure of the AIOM touch sensor in front section view

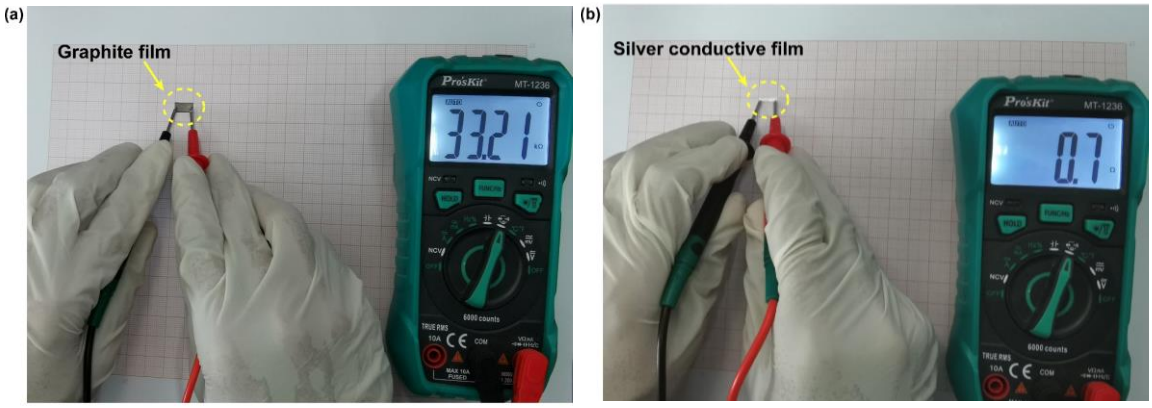

Fig. S2 Typical resistances of **a** graphite film and **b** silver conductive film. The resistance range of a typical graphite film was in the level of kilo ohms. The resistance of a typical silver conductive film was relatively small in the range of several ohms

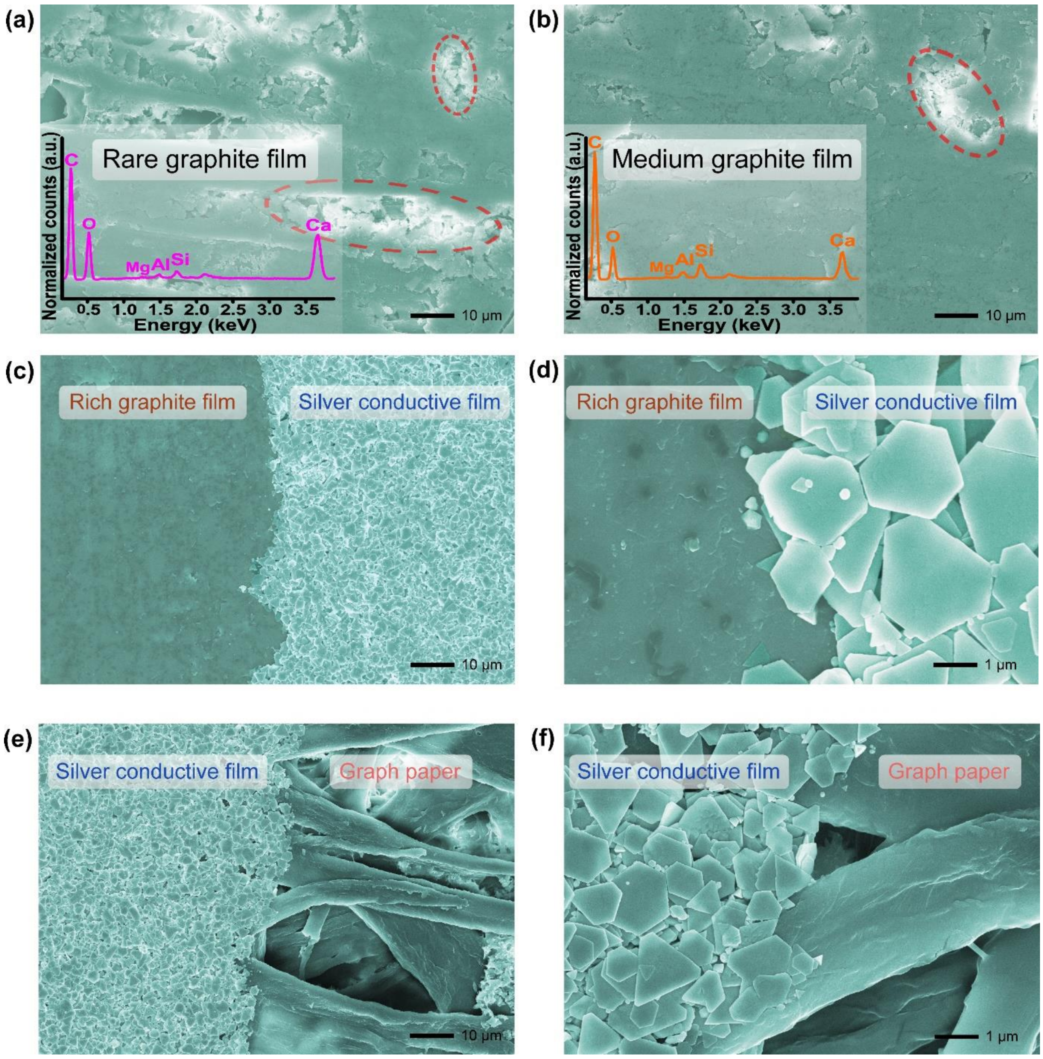

Fig. S3 Characteristics and detail morphologies of the materials. **a** The field emission scanning electron microscope (FESEM) image of the rare graphite film prepared by 8B pencil. Bottom left inset was the energy spectra of the rare graphite film. The red dotted circles point out areas where graphite is rare on graph paper. **b** The FESEM image of the medium graphite film prepared by 8B pencil. The corresponding energy spectrum was placed in the bottom left corner. The red dotted circle points out area where graphite is rare on graph paper. **c**, **d** The FESEM images of the junction of the rare graphite film and silver conductive film. The rich graphite

film was on the left and the silver conductive film was on the right, which showed the clear boundary between the rich graphite film and the silver conductive film. **e**, **f** The FESEM images of the junction of the silver conductive film and the graph paper. The silver conductive film was on the left and the graph paper was on the right, which showed the clear boundary between the silver film and the graph paper

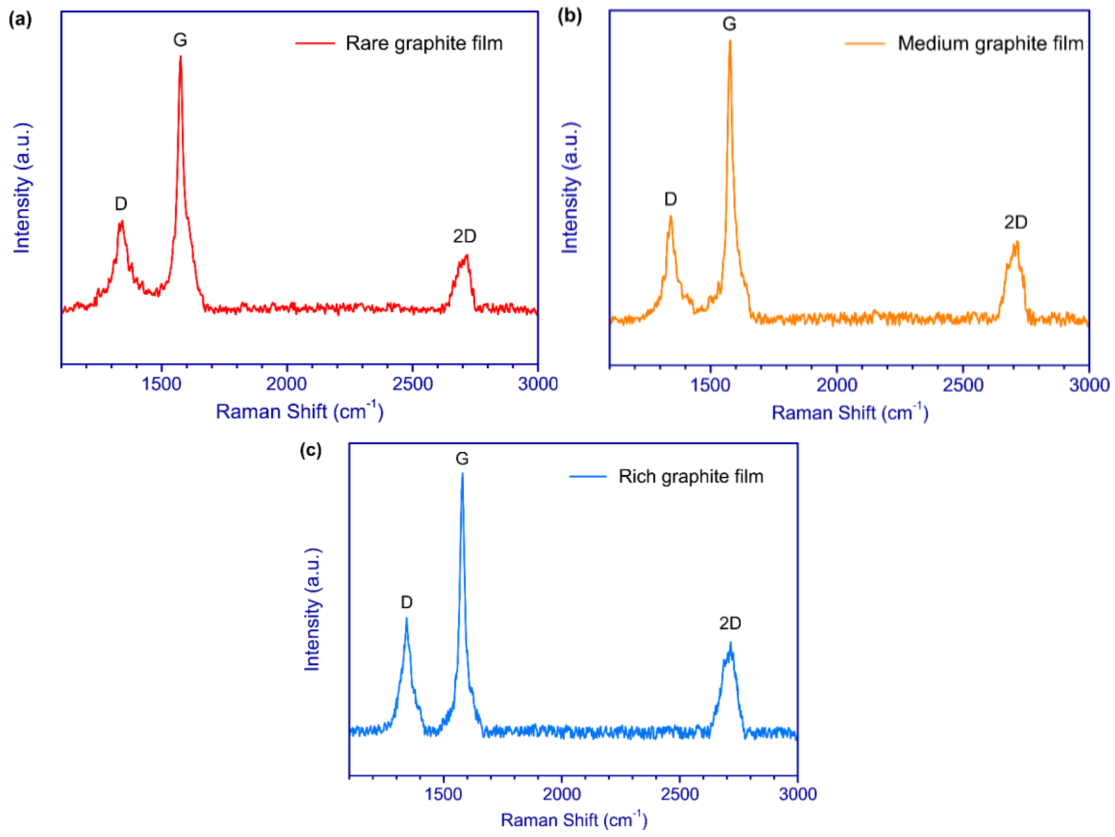

Fig. S4 Raman spectrum of graphite films with different carbon content. **a** Raman spectrum of the rare graphite film. **b** Raman spectrum of the medium graphite film. **c** Raman spectrum of the rich graphite film

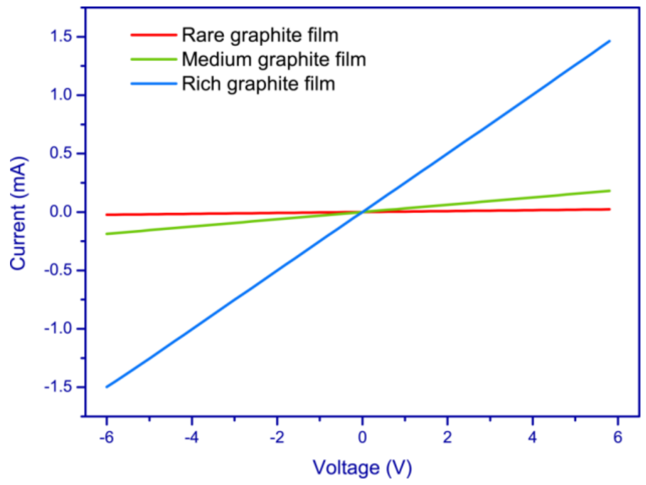

Fig. S5 Current-voltage characteristic curves of the graphite films with differernt carbon content. The length and the width of the graphite film were 10 mm and 5 mm, respectively. It could be found that the graphite films were resistive-type elements. The resistance of the rare graphite film, medium graphite film, and rich graphite film was 256, 32, and 4 kΩ, respectively. It should be noted that the resistance of the graphite film could be effectively modulated by

pencil repeatedly drawing on the graph paper and eraser removing some of the graphite. Therefore, the graphite films with different carbon content were designed as the conductive signal transmission channel and the gradient resistance elements of the AIOM touch sensor in a certain resistance range.

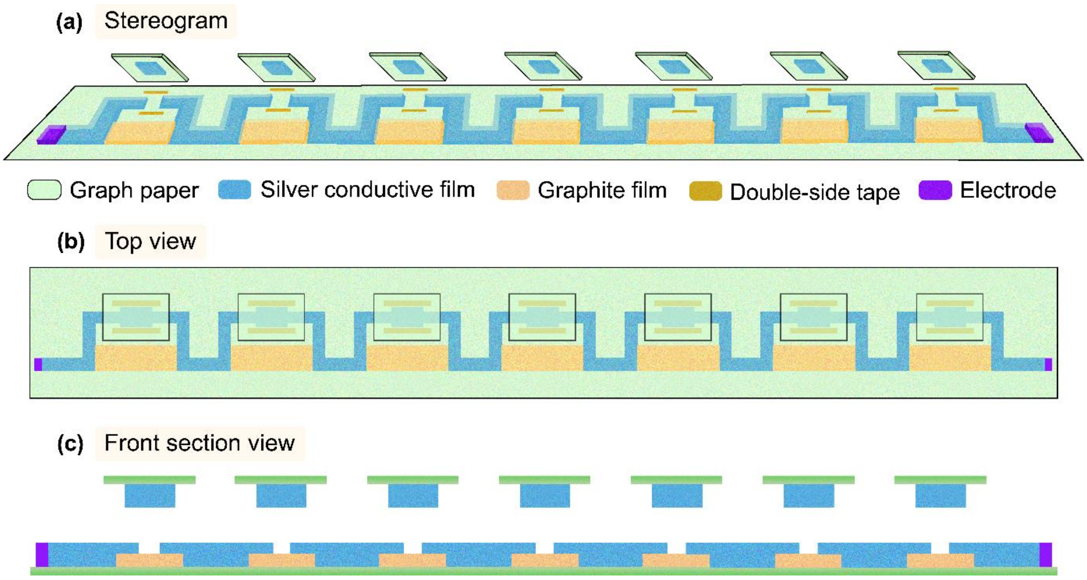

Fig. S6 Structure diagram of the AIOM touch sensor. **a** The stereogram of the breakdown structure of the AIOM touch sensor with seven gradient resistance elements and corresponding seven active touch buttons. **b** The simplified structure of the AIOM touch sensor in top view. The explanation of the various color symbols was between **a** and **b**. **c** The simplified breakdown structure of the AIOM touch sensor in front section view

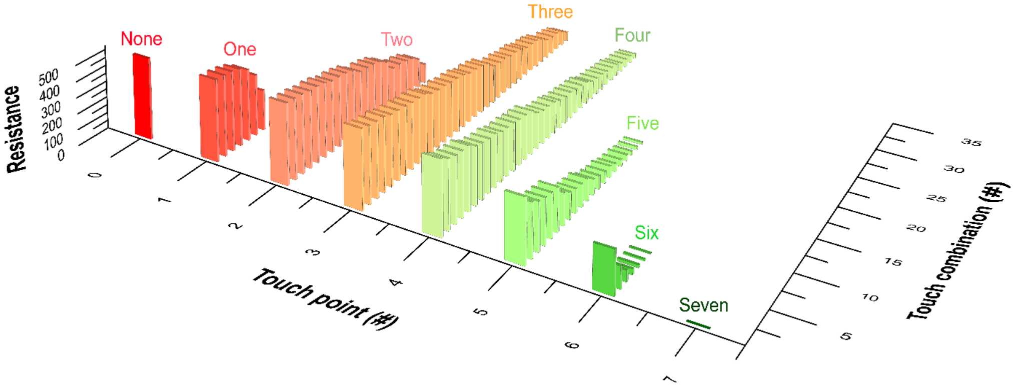

Fig. S7 Touch response resistance set of the AIOM touch sensor designed with seven active touch buttons. X-axis from left to right showed none-point touch, one-point touch, two-point touches, three-point touches, four-point touches, five-point touches, six-point touches, and seven-point touches in sequence. In the design scheme, there were a total of 128 response resistance values ranging from 30 to 508 kΩ. Each interval of the response resistance was about 4 kΩ. For one-point touch, there were a total of 7 touch positions and corresponding 7 response resistances. For two-point touches, there were a total of 21 touch combinations and corresponding 21 response resistances. For three-point touches, there were a total of 35 touch combinations and corresponding 35 response resistances. For four-point touches, there were a total of 35 touch combinations and corresponding 35 response resistances. For five-point touches, there were a total of 21 touch combinations and corresponding 21 response resistances. For six-point touches, there were a total of 7 touch combinations and corresponding 7 response resistances. For seven-point touches, there was a total of 1 touch combination and

corresponding 1 response resistance. The result indicated that the response resistance was highly regional differentiated to all touch cases. Therefore, the AIOM touch sensor based on the structure of two electrodes and the gradient resistance elements could fully promote wider detection and recognition range of mechanical stimulations and provide richer possibilities and practicability for artificial intelligence-assisted human-machine interactive applications.

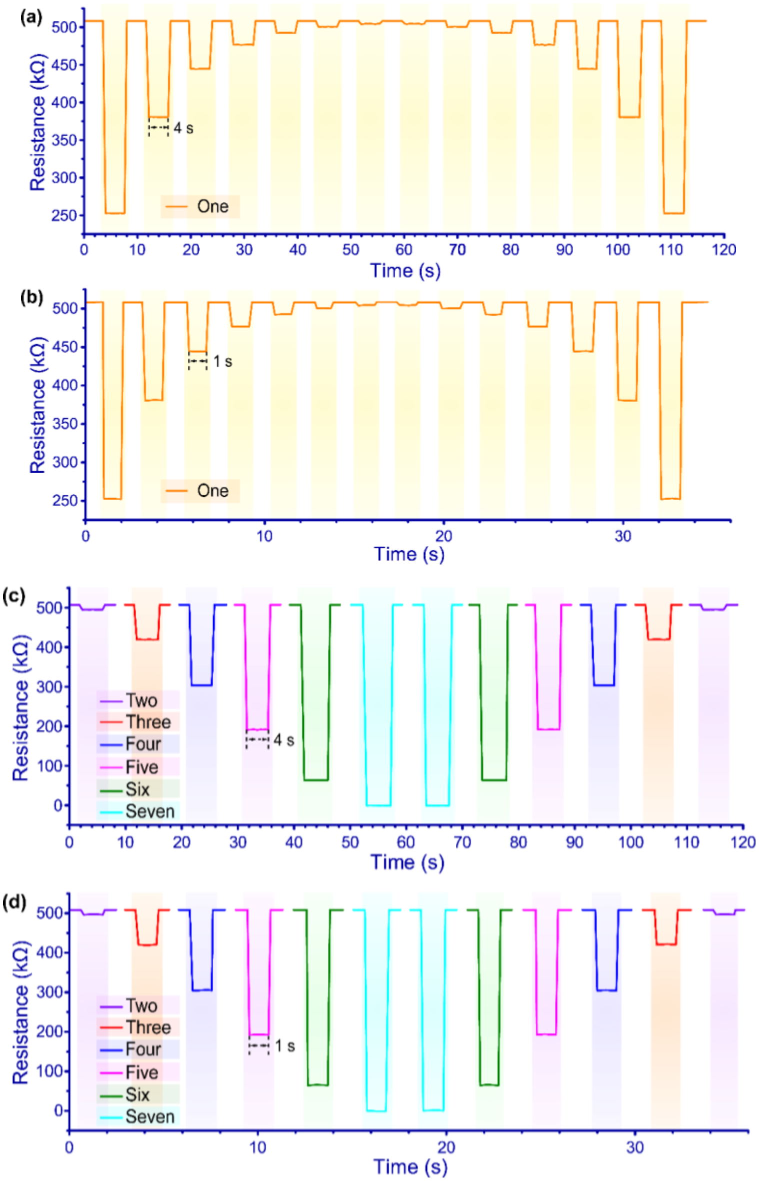

Fig. S8 Spatiotemporally dynamic stimulations based on one-point touch and multipoint touches circularly applying on the AIOM touch sensor. Response resistance of the AIOM touch sensor for one dynamic mechanical stimulations and information fusion of dynamic mechanical stimulations based on multipoint touch positions with the different lasting time of **a**, **c** 4 s and **b**, **d** 1 s. The results demonstrated that the AIOM touch sensor had high robustness and stability in operation. The results further indicated that the mechanosensitive signals were highly regional differentiated to all cases of dynamic mechanical stimulations, and the AIOM touch sensor well realized the establishment and judgment of spatiotemporally dynamic logic with multiple combinations of touches

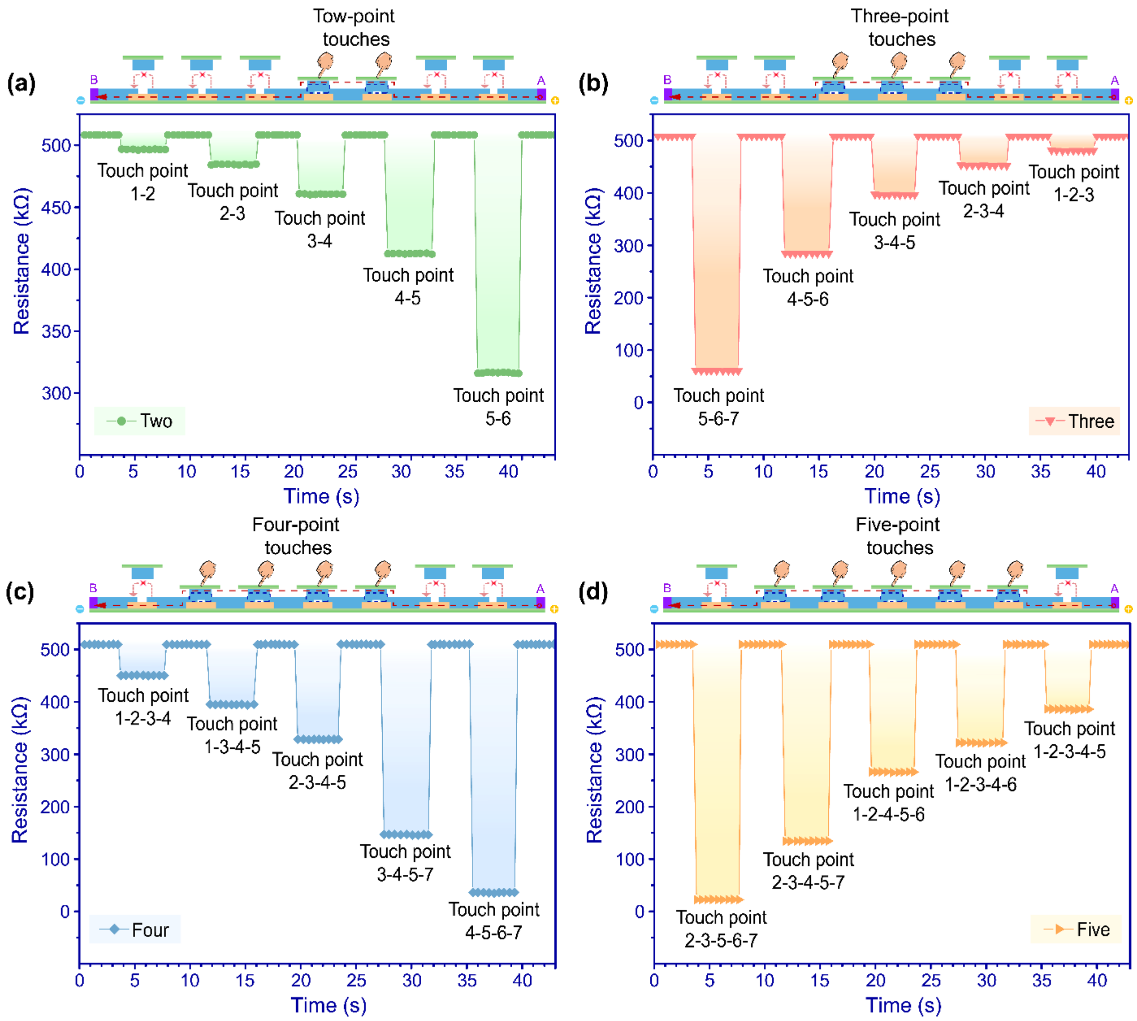

Fig. S9 Response resistance of the linear AIOM touch sensor for pressing different touch positions and different numbers of active touch buttons for playing piano. **a-d** Two, Three, Four and Five mechanical stimulations on the AIOM touch sensor and response resistance of mechanical stimulations

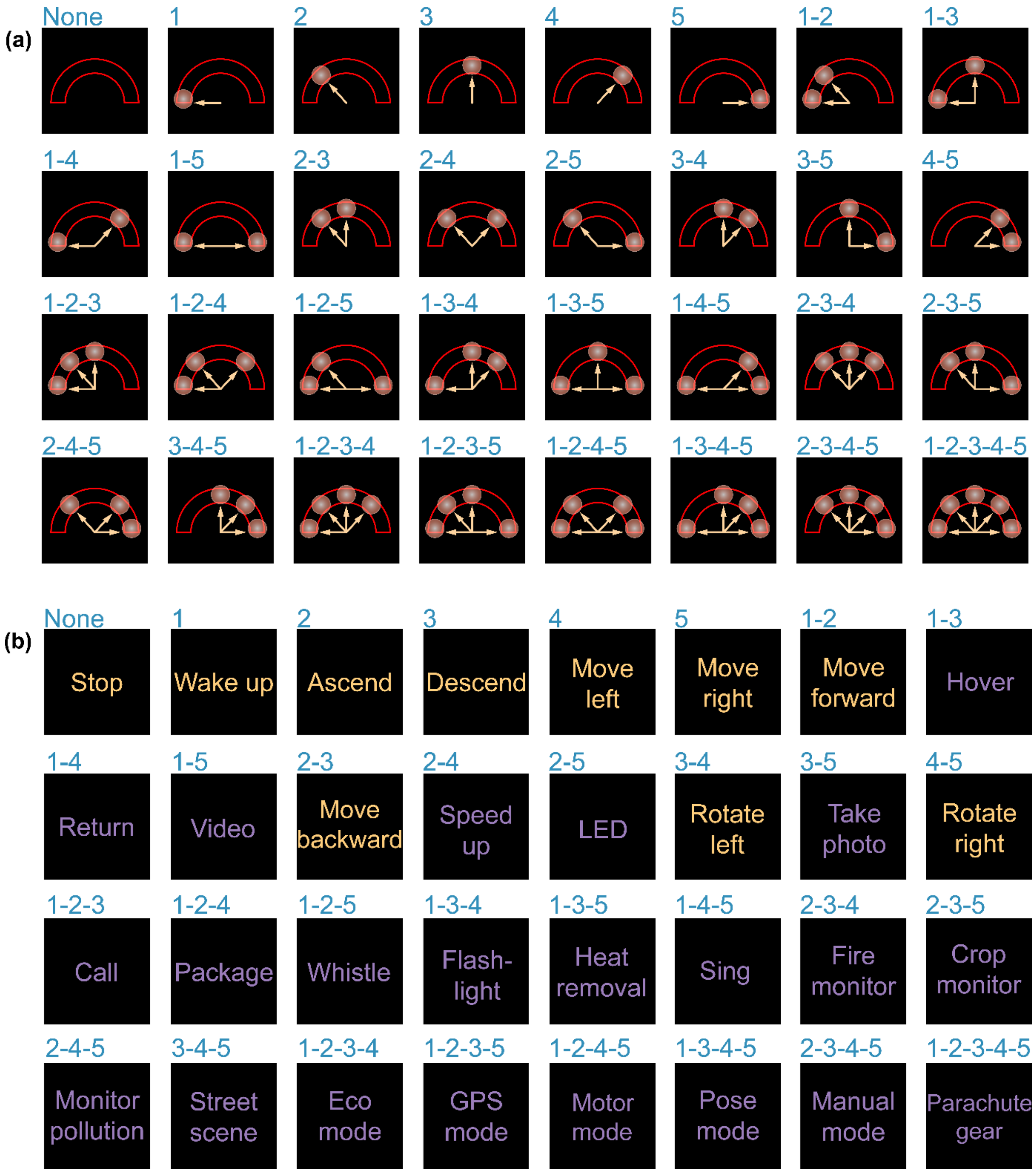

Fig. S10 The programmable touch operation platform and drone handling functions. **a** 32 touches of interaction logic method controlling the drone by the AIOM touch sensor. The red area represented the designed AIOM touch sensor location, and the yellow bright spot represented the corresponding trigger button pressing on the multipoint touch sensor. There were five touch locations on a circular AIOM touch sensor. The number of bright yellow spots in the figure indicated the amount of active touch buttons pressed. **b** The drone operation equipped by the AIOM touch sensor was conceptually implemented with a wealth of practical control functions. The yellow font indicated the functions that have been used in the design application of supplementary movie 3, and the purple font indicated the pending function combination in the future design

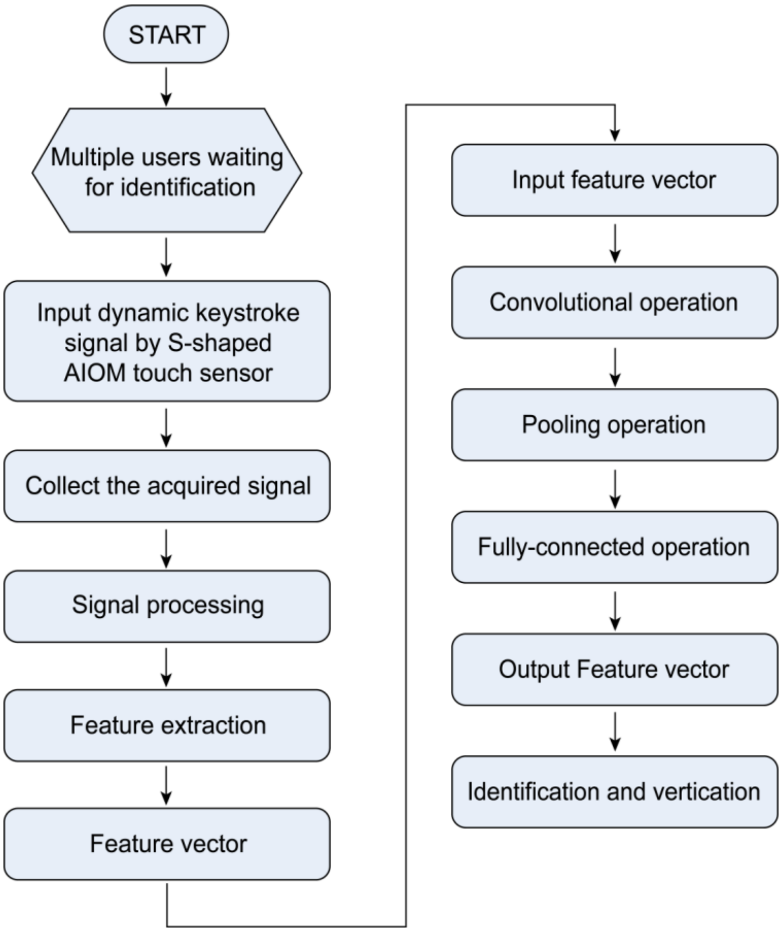

Fig. S11 Signal processing flowchart of the artificial neural network (ANN) algorithm procedure of proposed keystroke dynamics-based multiple users' identification and verification system based on the S-shaped AIOM touch sensor

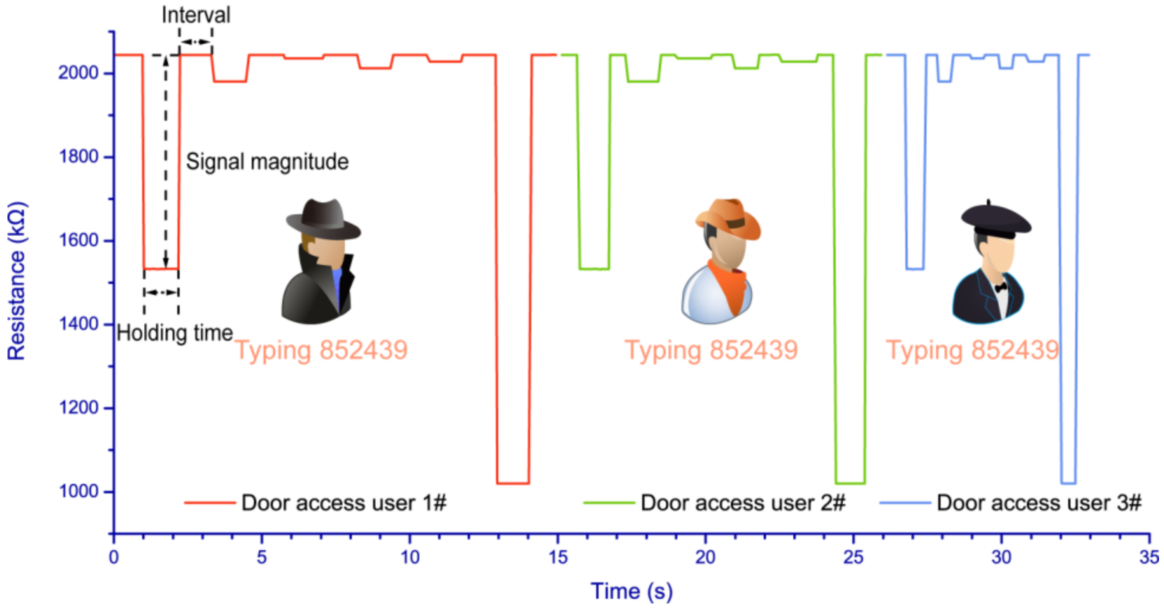

Fig. S12 Response resistance for the users' dynamic behavioral features of multiple touches. The user entered the same password "852439" in front of the door access. The key holding time and the interval between keys for the first user were both about 1 s. The key holding time for the second user was about 1 s, and the interval between keys for the second user was about 0.5 s. The key holding time for the third user and the interval between keys for the third user were both approximately 0.5 s

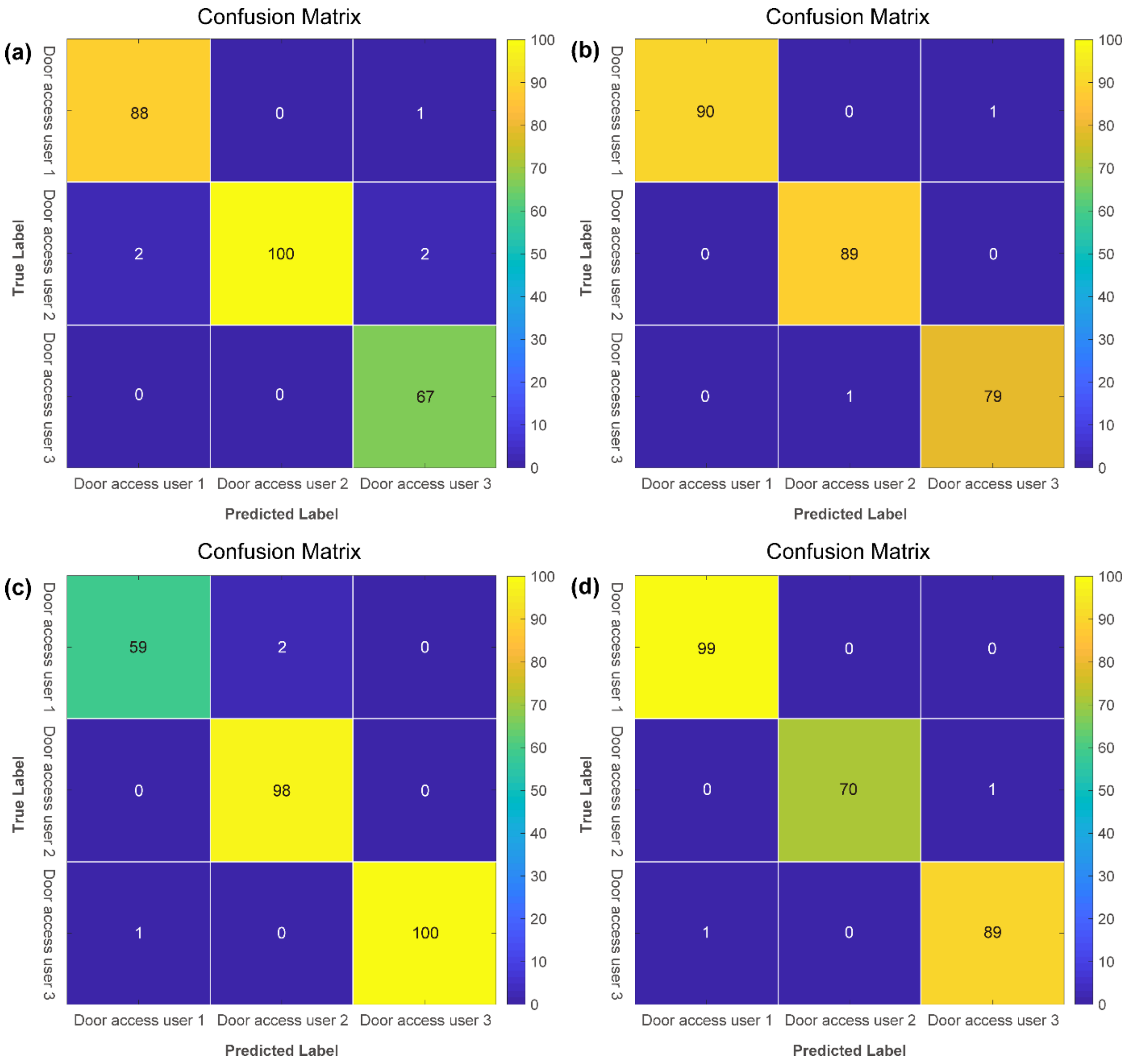

Fig. S13 Accuracy confusion matrix of augmented user verification system based on the S-shaped AIOM touch sensor. We tested the ANN algorithm based on the S-shaped AIOM touch sensor for four different test datasets of user 1, user 2, and user 3, respectively, with a total of 260. Diagrams of a, b, c, and d in the test datasets indicates 90, 100, 70; 90, 90, 80; 60, 100, 100; 100, 70, 90 for user 1, user 2 and user 3, respectively. The confusion matrices (Fig. S13) showed that, during the identification and verification process of the user access control, the ANN algorithm based on the S-shaped AIOM touch sensor achieved an accuracy of 98.1%, 99.2%, 98.8%, 99.2% for the biometrics application with keystroke dynamics respectively. Overall, the confusion matrices indicated that the ANN algorithm achieved an accuracy of over 98% for identification and verification
